# Treatment of ANCA-Associated Glomerulonephritis Complicated by Bacteremia and Vertebral Osteomyelitis: A Challenging Medical Situation

**DOI:** 10.1155/crin/4392221

**Published:** 2025-10-15

**Authors:** Bahjat Azrieh, Abdullah Thayyil, Mohamed Fawzi Mudarres, Nada Youssef, Naga Sumanth Gopireddy, Douglas Somers, Melissa Swee

**Affiliations:** ^1^Department of Internal Medicine, University of Iowa Hospitals and Clinics, Iowa City, Iowa, USA; ^2^Department of Pathology, University of Iowa Hospitals and Clinics, Iowa City, Iowa, USA; ^3^The University of Texas MD Anderson Cancer Center, Houston, Texas, USA; ^4^VA Iowa City Healthcare System, Iowa City, Iowa, USA

**Keywords:** AKI, ANCA, CKD, crescentic glomerulonephritis, discitis, disseminated zoster, epidural abscess, glomerulonephritis, infection, microscopic polyangiitis

## Abstract

Although the prognosis for patients with ANCA-associated vasculitis (AAV) has improved with modern immunosuppressive drugs, treatment-related complications continue to contribute significantly to morbidity and mortality. Infections, in particular, pose a major risk. Older age, high disease activity at diagnosis, and use of potent immunosuppressants are the most important prognostic factors. Older age is independently associated with mortality, severe renal failure, pulmonary hemorrhage, and relapse. This case highlights the challenge of balancing effective immunosuppression with the associated increased risk of infection. A 77-year-old male treated for MPO-ANCA–positive crescentic glomerulonephritis developed severe complications, including bacteremia, osteomyelitis, and disseminated herpes zoster, ultimately resulting in septic shock. Emerging therapies such as avacopan and predictive tools such as the Death in ANCA Glomerulonephritis–Estimating the Risk (DANGER) score may help clinicians better navigate these complex scenarios by guiding treatment intensity and minimizing risks.

## 1. Introduction

Antineutrophil cytoplasmic antibody (ANCA)–associated vasculitis (AAV) is a systemic autoimmune disease characterized by necrotizing inflammation of small- to medium-sized vessels. The presence of myeloperoxidase (MPO) or Proteinase 3 (PR3) autoantibodies facilitates the recruitment and activation of neutrophils, leading to endothelial damage [1, 2]. Pauci-immune necrotizing and crescentic glomerulonephritis is a frequent component of AAV. AAV predominantly affects individuals over 50 years old, where age-related comorbidities exacerbate treatment challenges [3, 4].

Immunosuppressive therapies are used to induce remission, maintain remission, and treat relapses. These agents have transformed AAV from a rapidly fatal disease to one characterized by periods of remission and relapse. However, their associated risks, including opportunistic infections, remain a significant concern [4, 5]. Infections constitute a leading cause of mortality, especially during the early phases of remission induction, with elderly patients being particularly vulnerable [3, 5].

Severe infections are common in AAV, with pneumonia, sepsis, and urinary tract infections being the most frequently reported [6]. These infections not only contribute to significant morbidity but are also associated with permanent organ damage and high mortality [5, 7]. While opportunistic infections such as *Pneumocystis jirovecii* constitute a smaller proportion of infections (6%), their impact is substantial due to the high degree of immunosuppression required for AAV management [5].

Here we report a case of MPO-ANCA–positive crescentic glomerulonephritis in a 77-year-old male, whose immunosuppressive therapy was complicated by severe bacterial and viral infections, ultimately leading to septic shock. This case highlights the challenges of balancing effective immunosuppression with the increased risk of life-threatening infections.

## 2. Case Presentation

A 77-year-old man with a history of atrial fibrillation on apixaban, hypertension, and antiglomerular basement membrane (GBM) disease 22 years prior, leading to chronic kidney disease (CKD) Stage 3a, was admitted following a burn injury. On admission, his vital signs included a temperature of 36.7°C, blood pressure of 152/88 mmHg, heart rate of 63 bpm, respiratory rate of 18 breaths per minute, and oxygen saturation of 96% in room air. During his hospitalization, his kidney function worsened from baseline. He was readmitted 2 months later with worsening renal function, fatigue, and volume overload.

**Figure 1:**
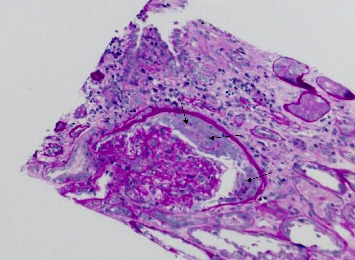
A glomerulus with cellular crescents (PAS stain, 200x magnification).

A kidney biopsy performed during this admission demonstrated ANCA-associated, pauci-immune, crescentic glomerulonephritis with IgA deposits, moderate to severe activity and chronicity, and moderate to severe arteriolar hyaline sclerosis (Figure 1). The presence of IgA deposits raised concern for an infectious trigger, though these were ultimately deemed an epiphenomenon. Anti-GBM antibodies were negative, and MPO-ANCA titers were markedly elevated at 1:2560, raising the possibility of microscopic polyangiitis with crescentic glomerulonephritis. Based on the Death in ANCA Glomerulonephritis–Estimating the Risk (DANGER) score, the patient's risk of mortality was calculated as 22.7% at 1 year, 39.4% at 3 years, and 50.4% at 5 years.

**Figure 2:**
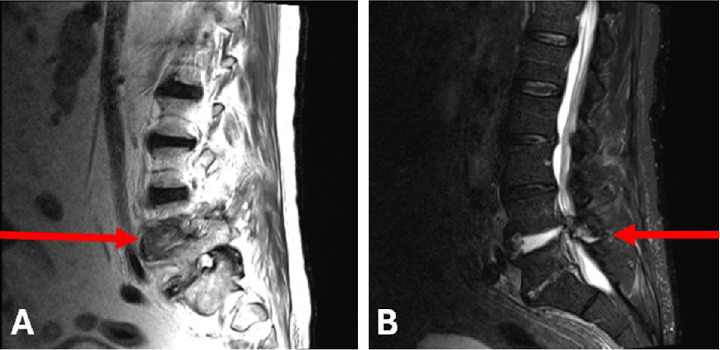
T1 and T2 STIR images of the MRI of the lower back show extensive discitis secondary to *Enterobacter cloacae* infection.

The patient received high-dose IV methylprednisolone followed by a prednisone taper and one dose of rituximab for induction therapy. One week later, he was readmitted for falls, worsening kidney function, and lower limb edema, and he was treated with IV diuretics for volume overload. Further evaluation with lumbar MRI demonstrated L4/L5 discitis and osteomyelitis, with a possible epidural abscess (Figure 2).

**Figure 3:**
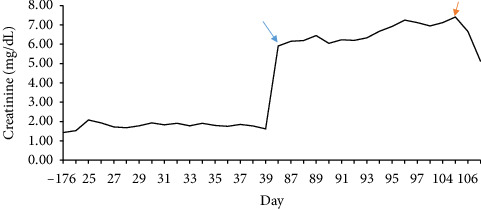
Blue arrow indicates AKI. Red arrow indicates the start of dialysis.

Blood cultures grew *Enterobacter cloacae*, and the patient was treated with a 6-week course of ertapenem followed by a transition to oral ciprofloxacin for 2 weeks. The patient's clinical course was further complicated by disseminated herpes zoster, for which he was treated with IV acyclovir. His second dose of rituximab was withheld due to ongoing infections. Despite treatment, his CKD progressed to end-stage kidney disease (ESKD), necessitating hemodialysis initiation (Figure 3). Unfortunately, his condition continued to decline, and he ultimately succumbed to refractory septic shock approximately 3 months after his initial diagnosis.

Note, IgA deposits were deemed as an epiphenomenon because of the presence of a different, more fundamental process happening, which is the ANCA glomerulonephritis. Infection in this patient happened after immunosuppressive treatment. He did not have an infection when the kidney biopsy was taken.

## 3. Discussion

AAV constitutes a heterogeneous set of small-vessel vasculitides that includes microscopic polyangiitis, granulomatosis with polyangiitis, and eosinophilic granulomatosis with polyangiitis. These share pathogenic, pathological, and clinical features involving inflammation of small- to medium-sized vessels, including capillaries, venules, arterioles, and small arteries [1, 2]. Approximately 90% of patients have autoantibodies to either MPO or to PR3 [8].

Renal manifestations range from rapidly progressive glomerulonephritis (RPGN) to an indolent, relapsing course, often leading to glomerulosclerosis. The hallmark glomerular lesion in AAV is focal necrotizing glomerulonephritis with crescent formation and minimal or absent glomerular immunoglobulin staining [9, 10]. Subclinical glomerulonephritis may remain undetected, causing advanced renal failure by the time of definitive diagnosis [11, 12].

Immunosuppressive therapy with agents such as rituximab and cyclophosphamide, combined with corticosteroids, is the cornerstone of AAV treatment [13]. Rituximab, a monoclonal antibody targeting CD20, depletes B cells, thereby reducing autoantibody production [14, 15]. Cyclophosphamide, an alkylating agent, induces lymphocyte apoptosis and broad immune suppression. While both agents are effective for inducing remission, they carry significant risks of secondary immunodeficiency, including hypogammaglobulinemia and lymphopenia, which predispose patients to bacterial and opportunistic infections [5]. In this case, secondary immunodeficiency likely contributed to the patient's disseminated herpes zoster and bacterial osteomyelitis.

AAV patients experience higher infection rates than those with other autoimmune diseases, such as lupus nephritis, under similar immunosuppressive regimens. A study comparing these populations found severe infections in 53% of AAV patients compared to 33% in lupus nephritis patients. The reliance on cyclophosphamide and the systemic nature of AAV, often involving both renal and pulmonary systems, may explain this discrepancy [4]. Opportunistic infections, although less common, remain a critical concern due to their association with profound immunosuppression [16].

The DANGER score has emerged as a valuable tool for predicting mortality risk in AAV, incorporating factors such as age, renal function, and disease severity [1]. This patient's DANGER score indicated a high risk, with an estimated 22.7% likelihood of death within 1 year, increasing to 39.4% at 3 years and 50.4% at 5 years. Risk stratification through such tools can guide treatment intensity, balancing disease control with the minimization of treatment-related complications [5].

Avacopan, a complement C5a receptor antagonist, represents a promising steroid-sparing alternative for AAV management. Early studies indicate that avacopan can maintain disease remission while reducing corticosteroid-associated adverse effects, including infections [17]. Avacopan reduces glucocorticoid exposure and toxicity. However, a definitive reduction in serious infection rates has not been demonstrated.

This case underscores the critical need for balancing immunosuppression to achieve disease control while minimizing the risk of life-threatening infections. Tailored regimens, including lower-dose corticosteroids or reduced exposure to cyclophosphamide, may mitigate risks without compromising efficacy. Data from the PEXIVAS trial support this approach, showing that reduced-dose corticosteroids were noninferior for death or ESKD, with a statistically significant reduction in serious infections [7].

AAV patients, like the subject of this case, represent a particularly vulnerable group requiring individualized care. This case highlights the importance of vigilant monitoring for secondary immunodeficiency, proactive infection prophylaxis, and the integration of predictive tools to guide therapy intensity. Advances in biomarkers and targeted therapies hold promise for improving outcomes while minimizing treatment-related complications.

## 4. Conclusion

This case highlights the complexity of managing AAV in elderly patients with multiple comorbidities. While immunosuppressive therapy is essential for disease control, it requires risk assessment and infection prevention strategies. Prognostic tools such as the DANGER score and novel therapies such as avacopan may improve outcomes by reducing treatment-related complications. Future research should focus on refining these strategies and expanding their application to high-risk populations.
